# Supplementary material for: Sulforaphane Inhibits Exhaustive Exercise-Induced Liver Injury and Transcriptome-Based Mechanism Analysis
Source: Nutrients. 2023 Jul 20;15(14):3220. doi: 10.3390/nu15143220 (PMC10386178; doi:10.3390/nu15143220)
Supplement: Supplementary file 1 [file nutrients-15-03220-s001.zip › Table S1.docx]

**Table S1 Primers for qRT-PCR**

| **Gene** | **Primer** | **Sequence** | **Product (bp)** |
| --- | --- | --- | --- |
| Gapdh | Forward  Reverse | TGTTTGTGATGGGTGTGAACC  AGTGGATGCAGGGATGATGT | 245 |
| Ppp1r3g | Forward  Reverse | TACTTTCCCGTCTCCACAGC  CAACTGATCACTCGGCCAGA | 247 |
| Crtam | Forward  Reverse | GGTATCCTGCTGCTCACACT  CCTGGGAAGATGTCTCCTCG | 178 |
| Tas1r2 | Forward  Reverse | CCTGCCCATCCTCAAAGACT  CTTGTCTCGCAGCTTGTCG | 160 |
| Tex35 | Forward  Reverse | TCCCAAGCCATGATCACAGA  TAGAAAGCAGCTCCAGACGA | 227 |
| Wnt6 | Forward  Reverse | GTGGACTTCGGGGATGAGAA  CCATGGCACTTACACTCGGT | 152 |
| Fbp2 | Forward  Reverse | GCTCTTGGAGAATTCGTGCT  CTCTTCTGGTTGGCTGGGTA | 245 |
| Nr4a2 | Forward  Reverse | CACTTGTGAGGGCTGCAAAG  AACCATCCCAACAGCTAGGC | 154 |
| Gm11525 | Forward  Reverse | GCGTGAGACTGAGTAACTGC GACTGTACCGTTGTCTTGGC | 216 |
| Hdc | Forward  Reverse | AATGTGCAGCCTGGATACCT  AGAGCAGGATAGTAGGCGTG | 155 |
| Gtse1 | Forward  Reverse | GTCTGCCCTGTTCCTCTCAG  TTTCGTCCTCTGAATGCTGG | 191 |
