# Supplementary figures and images for: Sulforaphane Inhibits Exhaustive Exercise-Induced Liver Injury and Transcriptome-Based Mechanism Analysis
Source: Nutrients. 2023 Jul 20;15(14):3220. doi: 10.3390/nu15143220 (PMC10386178; doi:10.3390/nu15143220)

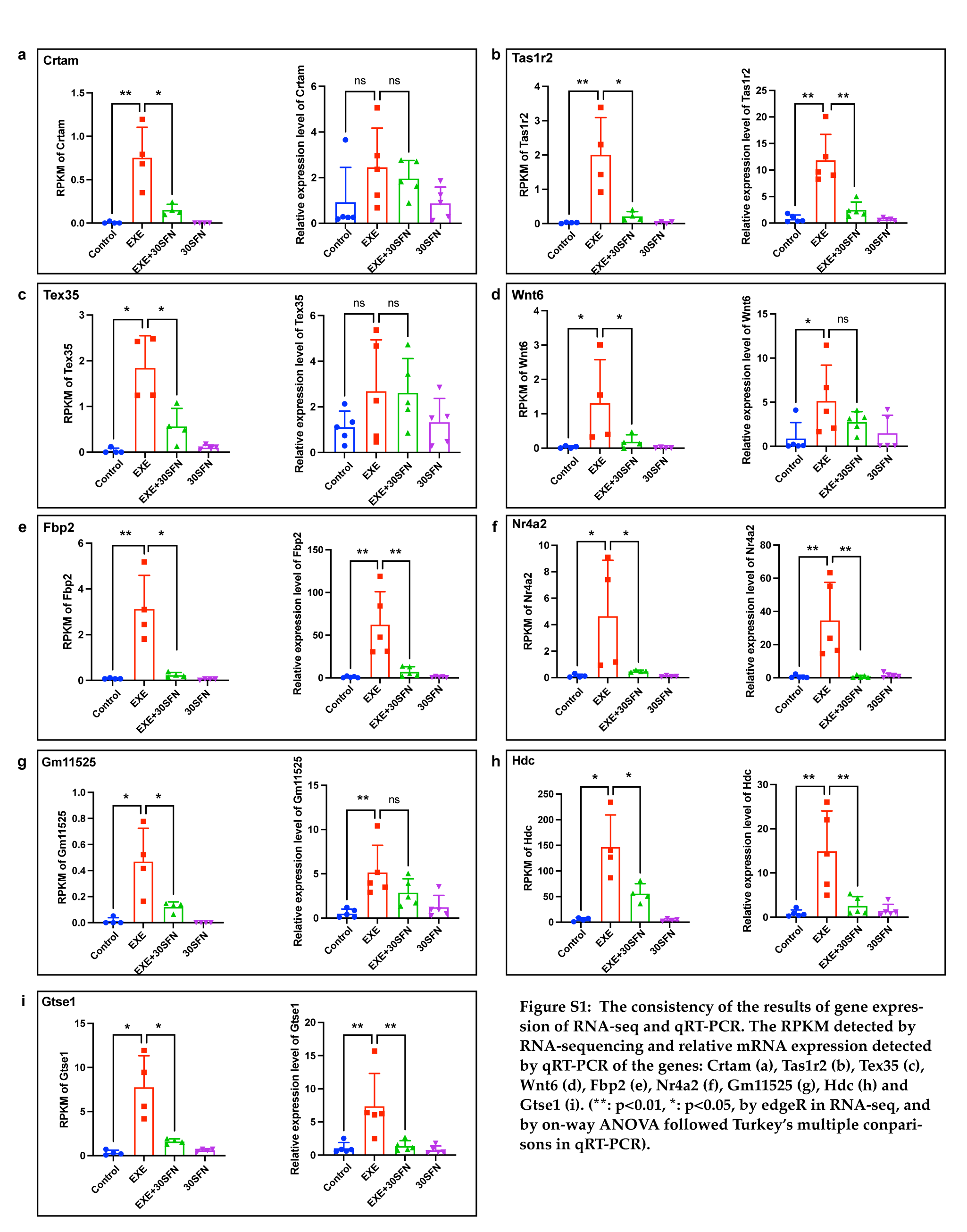

Supplement: Supplementary file 1 [file nutrients-15-03220-s001.zip › Figure S1.tif]
